# Valproic acid promotes the in vitro differentiation of human pluripotent stem cells into spermatogonial stem cell-like cells

**DOI:** 10.1186/s13287-021-02621-1

**Published:** 2021-10-29

**Authors:** Xiaotong Wang, Mengyuan Qu, Zili Li, Yuting Long, Kai Hong, Honggang Li

**Affiliations:** 1 Institute of Reproductive Health/Center of Reproductive Medicine, Tongji Medical College, Huazhong University of Science and Technology, Wuhan, 430030 China; 2 Wuhan Tongji Reproductive Hospital, Wuhan, 430013 China; 3 Department of Urology, Peking University Third Hospital, Beijing, 100191 China

**Keywords:** Azoospermia, Pluripotent stem cells, Valproic acid, Vitamin C, Spermatogonial stem cell, Wnt signaling pathway

## Abstract

**Background:**

Studying human germ cell development and male infertility is heavily relied on mouse models. In vitro differentiation of human pluripotent stem cells into spermatogonial stem cell-like cells (SSCLCs) can be used as a model to study human germ cells and infertility. The current study aimed to develop the SSCLC induction protocol and assess the effects of the developed protocol on SSCLC induction.

**Methods:**

We examined the effects of valproic acid (VPA), vitamin C (VC) and the combination of VPA and VC on the SSCLC induction efficiency and determined the expression of spermatogonial genes of differentiated cells. Haploid cells and cells expressed meiotic genes were also detected. RNA-seq analysis was performed to compare the transcriptome between cells at 0 and 12 days of differentiation and differently expressed genes were confirmed by RT-qPCR. We further evaluated the alteration in histone marks (H3K9ac and H3K27me3) at 12 days of differentiation. Moreover, the SSCLC induction efficiency of two hiPSC lines of non-obstructive azoospermia (NOA) patients was assessed using different induction protocols.

**Results:**

The combination of low concentrations of VPA and VC in the induction medium was most effective to induce SSCLCs expressing several spermatogonial genes from human pluripotent stem cells at 12 days of differentiation. The high concentration of VPA was more effective to induce cells expressing meiotic genes and haploid cells. RNA-seq analysis revealed that the induction of SSCLC involved the upregulated genes in Wnt signaling pathway, and cells at 12 days of differentiation showed increased H3K9ac and decreased H3K27me3. Additionally, two hiPSC lines of NOA patients showed low SSCLC induction efficiency and decreased expression of genes in Wnt signaling pathway.

**Conclusions:**

VPA robustly promoted the differentiation of human pluripotent stem cells into SSCLCs, which involved the upregulated genes in Wnt signaling pathway and epigenetic changes. hiPSCs from NOA patients showed decreased SSCLC induction efficiency and Wnt signaling pathway gene expression, suggesting that SSC depletion in azoospermia testes might be associated with inactivation of Wnt signaling pathway. Our developed SSCLC induction protocol provides a reliable tool and model to study human germ cell development and male infertility.

**Supplementary Information:**

The online version contains supplementary material available at 10.1186/s13287-021-02621-1.

## Introduction

Azoospermia referring to the complete lack of sperm in the ejaculate represents 10–20% cases of male infertility, and over 70% azoospermia patients are non-obstructive azoospermia (NOA) caused by the failure of spermatogenesis [1]. Spermatogenesis is a highly complex and precise process which involves spermatogonial stem cells (SSCs) differentiation into spermatocytes and following differentiation into functional mature spermatozoa [2]. The formation, maintenance and differentiation of SSCs secure the progress of spermatogenesis. In a variety of mouse models, the abnormalities of SSCs cause the absence of testicular germ cells and result in azoospermia, which is similar to that of the most severe form of NOA called Sertoli cell-only syndrome (SCOS) [3–7]. Currently, less is known about the etiology and pathogenesis of human azoospermia and therefore the development of diagnosis and therapy is faced with difficulties. In recent years, whole exome sequencing (WES) has been applied to identify genetic variants associated with NOA and several pathogenic variants were validated to possibly cause NOA by constructing the corresponding mouse models carrying the similar variants [8, 9]. Since the mouse and human reproductive systems are not identical and genes may have different functions or transmit disease through different modes of inheritance, caution is urged to draw conclusions on gene function and inheritance mode based on mouse models only [10]. Using human SSCs to study human azoospermia will provide direct and effective information. However, the acquisition and in vitro culture of human SSCs, as well as conducting experiments with these cells, are faced with ethical and technical problems.

The in vitro differentiation of human pluripotent stem cells into germ cell-like cells has opened a new pathway to directly study human germ cells and identify unique mechanisms in human reproduction [2]. A large number of germ cell-like cells can be obtained in a short time by this strategy. Easley et al. developed an “one-step” protocol to differentiate human induced pluripotent stem cells (hiPSCs) and human embryonic stem cells (hESCs) into SSC-like cells (SSCLCs). This protocol used the induction medium containing GDNF (20 ng/mL), b-FGF (1 ng/mL), various nutrients, lipid supplements and b-mercaptoethanol [11]. GDNF is mainly secreted by Sertoli cells and regulates cell fate of decisions of undifferentiated spermatogonial cells including SSCs [12, 13]. Lack of GDNF causes depletion of SSCs and high level of GDNF causes accumulation of undifferentiated spermatogonia [13]. b-FGF is also secreted by Sertoli cells and is another *bona fide* self-renewal factor for SSCs [14, 15]. These two cytokines, especially GDNF, played a crucial role in SSCLC induction, and the induction efficiency was low without GDNF [11]. Zhao et al. optimized Easley et al. protocol by culturing cells on gelatin without any feeder cells and replacing b-mercaptoethanol with vitamin C (VC) and bovine serum albumin (BSA) with xeno-free serum replacement [16]. VC in the induction medium circumvented the problem of cell death during differentiation [16]. Both protocols were able to differentiate human pluripotent stem cells into SSCLCs in their own hands, and the efficiencies were over 40% [11, 16], but the induction efficiencies varied among different stem cell lines [16].

In this study, we managed to modify SSCLC induction protocol based on the existing protocols to promote the SSCLC induction efficiency. We introduced valproic acid (VPA) into the SSCLC induction medium. VPA, a branched short-chain fatty acid, is one of the common HDAC inhibitors [17]. The known effect of VPA is increasing reprogramming efficiency through HDAC inhibition, which allows it to be used in iPSC formation [17]. VPA has additional activities beyond inhibition of HDACs [17]. Clinically, VPA is a widely and frequently used anti-epileptic drug since 1963, owing to its activities in inhibition of GABA transaminase and blocking voltage-gated sodium channels and T-type calcium channels [18, 19], and it has potential for the treatment of other neurological diseases, cancer and virus infection with unclear mechanisms [20–22]. VPA has been used to promote the differentiation of human stem cells into different types of somatic cells. It improved neural differentiation of human iPSCs [23], ESCs [24], mesenchymal stem cells [25] and adipose-derived stem cells [26], also hepatic and cardiomyocyte differentiation of human stem cells [27, 28]. We showed that VPA robustly elevated the SSCLC induction efficiency in the presence of GDNF and b-FGF, and the combination of low concentrations of VPA and VC achieved the highest SSCLC induction efficiency. Our SSCLC induction protocol was suitable to differentiate different hiPSC lines into SSCLCs. Using the model of differentiation of hiPSCs into SSCLCs, we found that Wnt signaling pathway was involved in SSCLC induction, and low SSCLC induction efficiency of NOA hiPSC lines might be associated with inactivation of Wnt signaling pathway.

## Methods

### Cell culture and differentiation

Human iPSC lines have been described previously [29, 30], including a normal cell line and two cell lines from unrelated NOA patients with unknown cause and testicular histology. The H1 ESC line was a gift from Professor Li Wang at State Key Laboratory of Cardiovascular Disease, Fuwai Hospital, Beijing and kept in our laboratory for study [31]. hiPSC lines and H1 ESCs were maintained in mTeSR1 medium (STEMCELL Technologies) at 37℃ and 5% CO_2_. The medium was changed every day. Cells were passaged every 3 to 4 days using 0.5 mM EDTA. An amount of 10 μM ROCK inhibitor Y-27632 (Selleck) was added into the medium for 24 h after every passage.

For hiPSCs and hESCs differentiation into SSCLCs, cells were firstly seeded on Matrigel (Corning)-coated 12 or 24-well plates in mTeSR1 medium containing 10 μM ROCK inhibitor, and the medium was replaced by SSCLC induction medium on the second day when cells reached 80%-90% confluence. The components of SSCLC induction medium were based on previous studies with some modifications [11, 16], which contained a-MEM, 3% KnockOut serum replacement, 1% GlutaMAX supplement, 1% 100 × Insulin-Transferrin-Selenium-X, 0.2% chemically defined lipid concentrate (all from Thermo Fisher Scientific), 20 ng/mL human GDNF, 1 ng/mL human b-FGF (all from Peprotech), 100–200 μg/mL VPA and/or 100–200 μg/mL VC (all from Sigma Aldrich). The concentrations of human GDNF, human b-FGF, VPA and VC varied with the purposes of experiments. The SSCLC induction medium was changed every day until the cells were ready for analysis.

### Flow cytometry

Cells were dissociated into single cells by Accutase (Thermo Fisher Scientific) and then fixed using 4% paraformaldehyde for 15 min at room temperature. Blocking and permeabilizing procedures referred to Zhao et al. protocol [16]. After the above processes, cells were suspended with 100 μL PBS containing 0.1% BSA and stained with PLZF monoclonal antibody-PE (1:200, Invitrogen, Mags.21F7) for 40 min under room temperature. Cells were then washed and resuspended with PBS containing 0.1% BSA, and the percentage of PLZF^+^ cells was detected by a DxFLEX flow cytometer (Beckman Coulter). For the detection of haploid cells, single cells were suspended with 300 μL cold PBS containing 10% FBS and then added with 700 μL cold ethanol. After stored at -20℃ overnight, cells were resuspended with PBS containing 100 μg/mL PI (Sigma Aldrich), 100 ng/mL RNase (CWBio, China) and 0.1% Triton X-100 for 20 min at room temperature. Cells were then washed and resuspended with PBS, and the percentage of haploid cells was detected by a DxFLEX flow cytometer. For the detection of apoptotic cells, single cells were stained with PI and Annexin V using a Annexin V-EGFP Apoptosis Detection Kit (KeyGEN BioTECH, China), and the percentage of apoptotic cells was detected by a DxFLEX flow cytometer.

### Immunofluorescence

Cells were fixed with 4% paraformaldehyde for 15 min at room temperature. After washed with PBS, cells were blocked and permeabilized with 5% BSA containing 0.3% Triton X-100 for 45 min at room temperature. Cells were then incubated with primary antibodies (Additional file 1: Table S1) at 4℃ overnight, followed by incubated with secondary antibodies at room temperature for 1 h (Additional file 1: Table S1). The nuclei were stained with DAPI. Cells were observed by a fluorescence microscope (Olympus).

### RT-qPCR

Total RNA of cells was extracted with TRIzol (Thermo Fisher Scientific) and then reverse transcribed into cDNA with HiScript III RT SuperMix (Vazyme, China) on a SimpliAmp Thermal Cycler (Thermo Fisher Scientific). RT-qPCR was conducted using ChamQ Universal SYBR qPCR Master Mix (Vazyme, China) on the StepOne Real-Time PCR system (Thermo Fisher Scientific). Primers used in this study were listed in Additional file 1: Table S2.

### Histone protein extraction and western blot

Cells (5 × 10^6^) were resuspended with 500 μL cold TEB (PBS containing 0.5% Triton X-100, 2 mM phenylmethylsulfonyl fluoride, 0.02% NaN_3_ and 5 mM sodium butyrate) and lysed on ice for 20 min. After centrifuged at 7000 × g for 10 min at 4℃, the pellet was suspended with 80 μL 0.2 N HCl at 4℃ overnight. Samples were centrifuged at 7000 × g for 10 min at 4℃ and the supernatant containing histone protein was neutralized with 2 M NaOH (10%). Histone protein was denatured with loading buffer and separated with 15% gradient SDS-PAGE and transferred to 0.22 μm PVDF membranes (Millipore). The membranes were blocked with 5% BSA and incubated with primary antibodies (Additional file 1: Table S1) at 4℃ overnight. The membranes were then incubated with secondary antibodies (Additional file 1: Table S1) at room temperature for 1 h and visualized with a BeyoECL Plus kit (Beyotime, China) on a ChemiDoc XRS + System (Bio-Rad).

### RNA-seq and data analysis

Total RNA was extracted using TRIzol from biological duplicates of cells from each group and used to construct RNA-seq libraries by TruSeq Stranded mRNA kit (Illumina). The libraries were sequenced using Illumina Novaseq 6000 platform. After removing the adaptor sequence and low-quality reads, clean reads were mapped to the human genome GRCh38/hg38. The aligned reads of genes were counted and normalized to evaluate gene expression as normalized counts per million. Significantly differentially expressed genes (DEGs) were those with false discovery rate (FDR) < 0.05 and |log_2_(fold change)|> 1. Gene ontology (GO) and pathways analyses were performed using the PANTHER classification system (http://www.pantherdb.org) [32].

### Statistical analysis

Data were presented as mean ± SD. Statistical analyses were conducted with One-way ANOVA using GraphPad Prism 9, and figures were also created with this software.

## Results

### VPA promoted the SSCLC induction efficiency

**Fig. 1:**
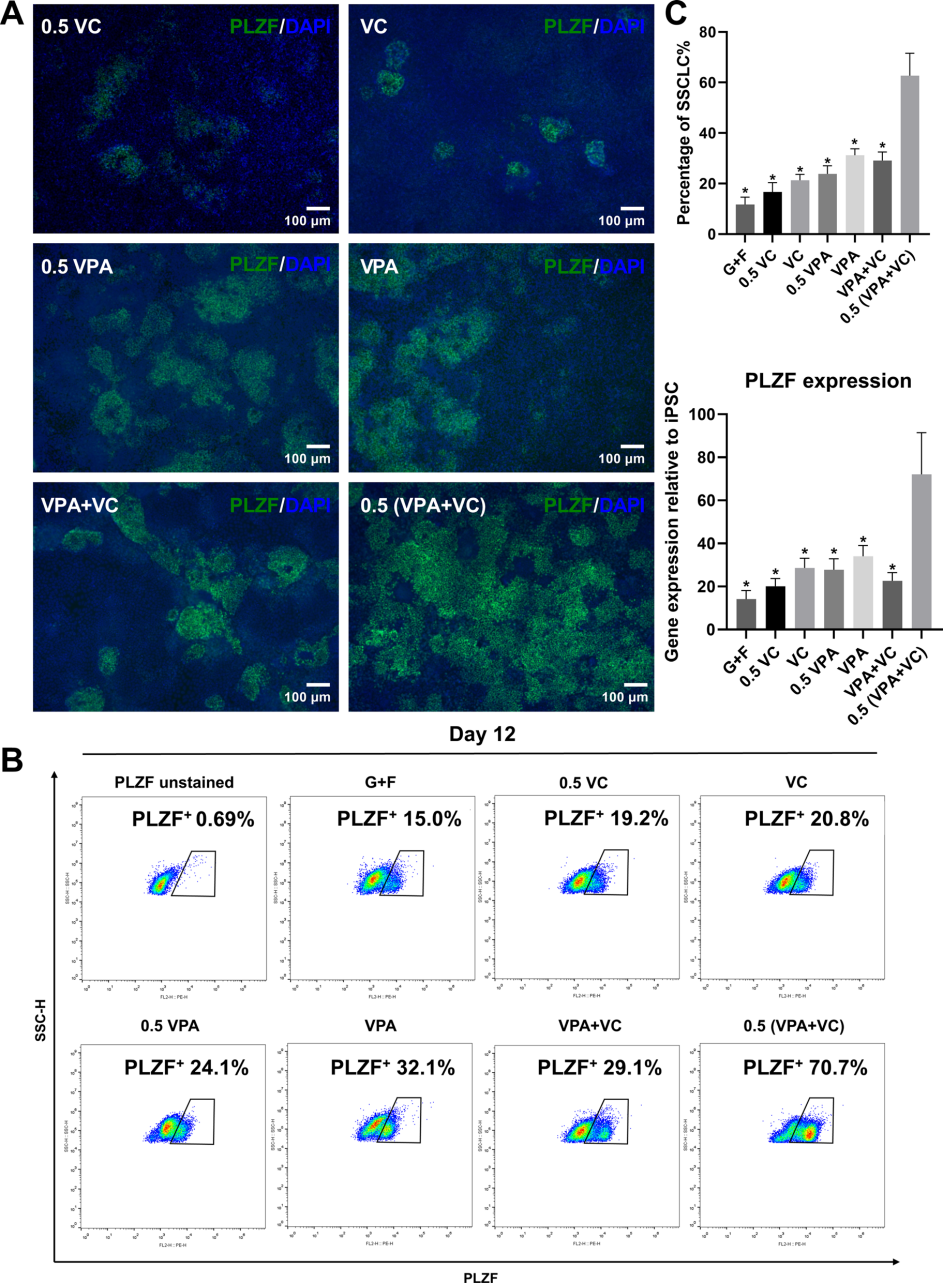
VPA promoted the differentiation of hiPSCs into SSCLCs. **A** Immunostaining of PLZF (green) of differentiated cells using SSCLC induction medium containing different concentrations of VPA and/or VC at 12 days of differentiation, and the nuclei were stained with DAPI (blue). **B** The percentage of PLZF^+^ cells, representing the SSCLC induction efficiency, was detected by flow cytometry at 12 days of differentiation. **C** The percentage of SSCLCs and the expression of *PLZF* (detected by RT-qPCR) in different groups, n = 3, *p < 0.05 when compared with 0.5 (VPA + VC)

We firstly differentiated hiPSCs into SSCLCs using the induction medium mainly containing human GDNF, human b-FGF and VC (VC group), and the SSCLC induction efficiency was determined by the percentage of PLZF^+^ cells as described earlier [16]. According to previous studies, the percentage of SSCLCs reached relatively high levels from 10 to 15 days of differentiation [11, 16]. Therefore, we detected the SSCLC induction efficiency at 12 days of differentiation. VC increased the SSCLC induction efficiency compared with the induction medium containing GDNF and b-FGF without VC (G + F group) or with half concentration of VC (0.5 VC group) (Fig. 1A–C, Additional file 3: Fig. S2 A). We also differentiated H1 ESCs into SSCLCs using different induction medium and observed the similar results to that of hiPSCs (Additional file 2: Fig. S1). We noticed that different cell lines showed different SSCLC induction efficiencies when used the same induction medium. The SSCLC induction efficiency of VC group in our hands was around 20%, and the induction efficiency could be improved. To achieve a higher induction efficiency, we considered to increase the concentrations of GDNF and b-FGF since these two cytokines have pivotal roles in regulating SSCs. A study reported that 20 and 40 ng/ml of GDNF could strongly promote the growth of mouse SSCs [12]. However, the SSCLC induction efficiency was not significantly changed when the concentrations of GDNF and b-FGF increased either alone or both (Additional file 3: Fig. S2), indicating that 20 ng/mL GDNF and 1 ng/mL b-FGF were sufficient in the induction medium.

We introduced VPA into the SSCLC induction medium to test whether VPA could elevate the SSCLC induction efficiency. The concentration of VPA used in previous studies to exert its effects on reprogramming and differentiation varied from 0.5 mM to 3.0 mM (72–432 μg/mL) [17, 33], and clinically relevant concentrations of VPA (0.5 mM or 1.0 mM) were sufficient to increase gene expression in neural differentiation [33]. We examined the effects of 100 and 200 μg/mL VPA (0.5 VPA group and VPA group, respectively) on the SSCLC induction efficiency. The SSCLC induction efficiency increased in 0.5 VPA and VPA groups compared with the G + F group, and was higher than the VC group; the high concentration of VPA was more effective (Fig. 1A–C). We further examined the effects of the combination of VPA and VC on the SSCLC induction efficiency. Interestingly, the combination of high concentrations of VPA and VC (200 μg/mL VPA and VC, VPA + VC group) did not achieve a higher SSCLC induction efficiency, but the combination of low concentrations of VPA and VC (100 μg/mL VPA and VC), that is, 0.5 (VPA + VC) group, greatly elevated the SSCLC induction efficiency (Fig. 1A–C). RT-qPCR confirmed the expression of *PLZF* in different groups, and *PLZF* expression was the highest in 0.5 (VPA + VC) group (Fig. 1C, p < 0.05). Additionally, the high concentration of VPA and the combination of VPA and VC did not increase cell apoptosis during differentiation compared with the VC group (Additional file 4: Fig. S3). VPA was able to promote the SSCLC induction efficiency in the presence of GDNF and b-FGF. Although SSCLCs accounted for a small fraction of differentiated cells in early days of differentiation (day 4 and day 8), we still observed a higher SSCLC induction efficiency in the 0.5 (VPA + VC) group than that in other groups (Additional file 5: Fig. S4). The combination of low concentrations of VPA and VC achieved the highest SSCLC induction efficiency in this study, which could be used as the developed SSCLC induction protocol.

**Fig. 2:**
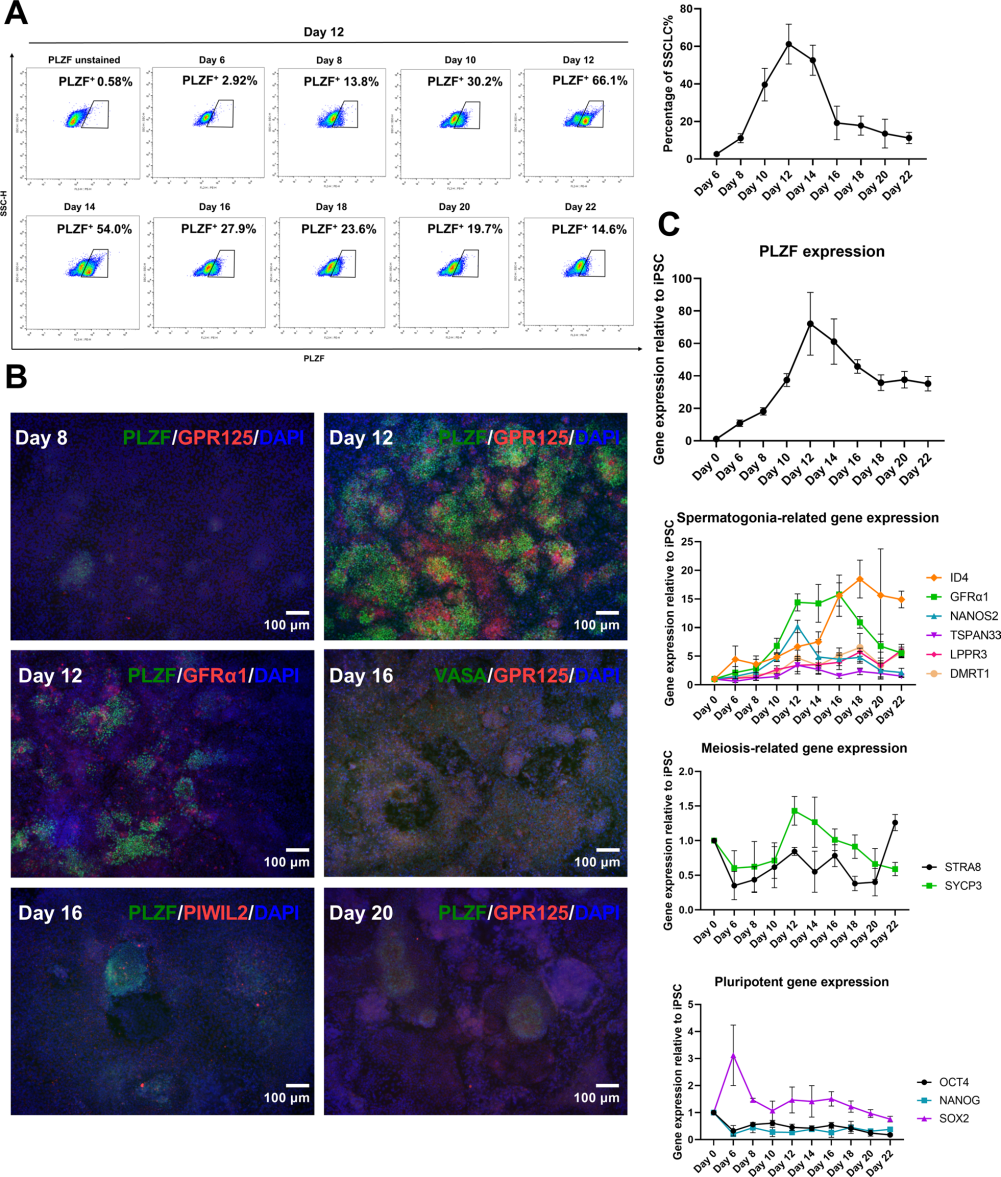
Gene expression and the SSCLC induction efficiency during SSCLC induction. **A** The percentage of PLZF^+^ cells at different days of differentiation detected by flow cytometry, n = 3. **B** Immunostaining of PLZF (green), GPR125 (red), GFRα1 (red), VASA (green) and PIWIL2 (red) of differentiated cells using developed SSCLC induction protocol at different days of differentiation, and the nuclei were stained with DAPI (blue). **C** The expression of genes related to SSC, meiosis and pluripotency at different days of differentiation detected by RT-qPCR, n = 3

To further clarify the effects of the combination of low concentrations of VPA and VC on the SSCLC induction process, we used the developed SSCLC induction protocol to differentiate hiPSCs for 22 days. At 6 days of differentiation, a very small portion of SSCLCs emerged; the percentage of SSCLCs continued to increase from day 8 to day 12; after day 12, the percentage of SSCLCs began to decrease (Fig. 2A). Immunofluorescence was used to stain the expression of SSC-related genes (*PLZF*, *GPR125*, *GFRα1*, *VASA* and *PIWIL2*) (Fig. 2B). The expression of PLZF and GPR125 was weak at day 8 and increased at day 12. The expression of GFRα1 was also strong at day 12. At day 16, the staining of PLZF, VASA and PIWIL2 still existed. At day 20, the expression of PLZF and GPR125 was decreased. The PLZF^+^ cells were GPR125, GFRα1 and PIWIL2 positive and some GPR125, GFRα1 and PIWIL2 positive cells did not express PLZF, indicating that PLZF^+^ cells were SSCLCs and the SSCLC induction system generated a mix of different types of cells. Moreover, PLZF^+^ cells had round and relatively small nuclei; they grew in an aggregated manner and formed clusters. RT-qPCR results showed the same expression tendency of *PLZF* as the results of flow cytometry and immunofluorescence, and the expression of spermatogonial genes (*ID4*, *GFRα1*, *NANOS2*, *TSPAN33*, *LPPR3* and *DMRT1)* also elevated during differentiation (Fig. 2C). In addition, except for *SOX2*, the expression of other pluripotent genes *OCT4* and *NANOG* decreased along with differentiation (Fig. 2C). These results showed that the SSCLC induction process elevated spermatogonial gene expression and repressed pluripotent gene expression.

### A relatively high concentration of VPA induced further differentiation

**Fig. 3:**
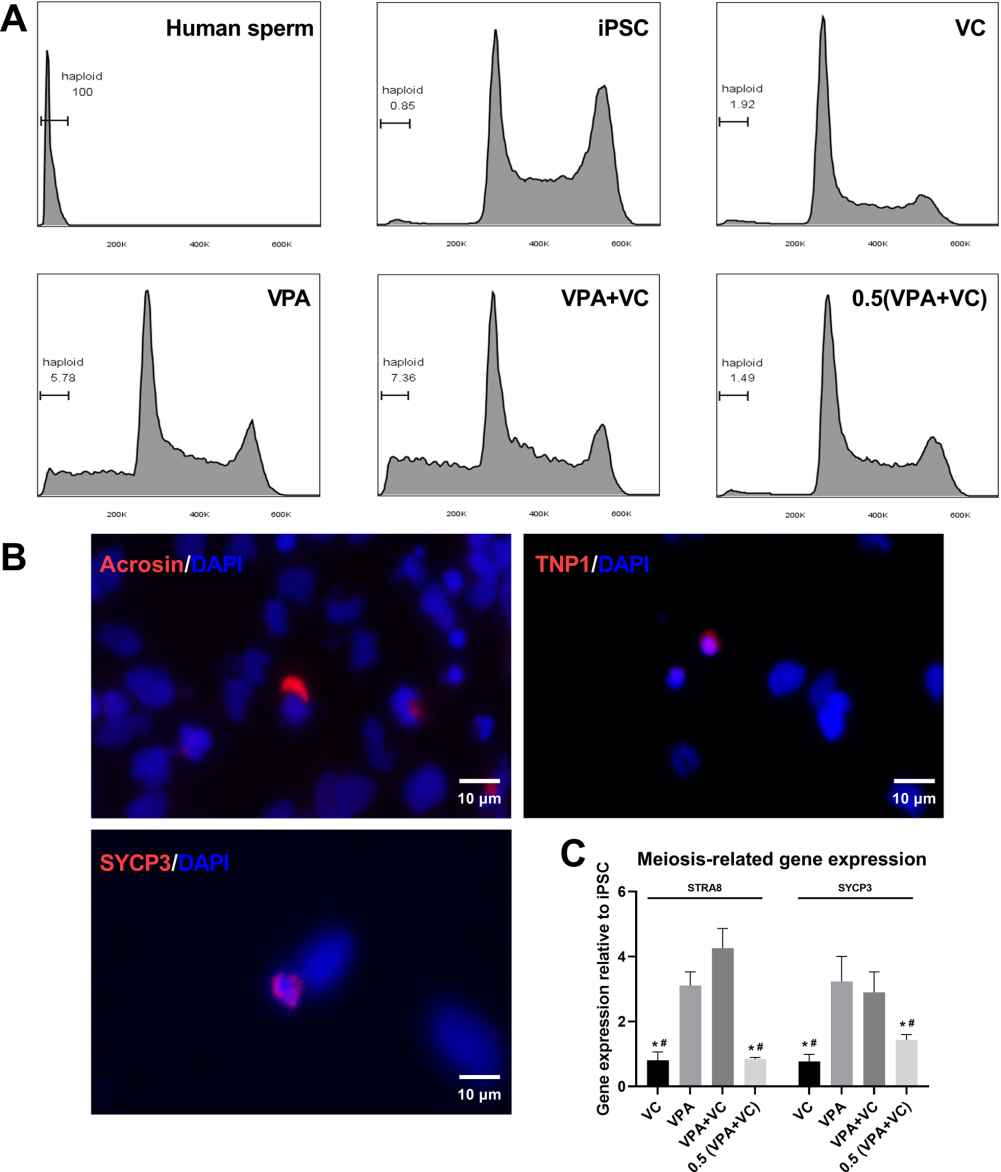
The high concentration of VPA induced further differentiation. **A** PI was used to stain DNA and the percentage of haploid cells at 12 days of differentiation was detected by flow cytometry. **B** immunostaining of Acrosin (red), TNP1 (red) and SYCP3 (red) of differentiated cells using developed SSCLC induction protocol at 12 days of differentiation, and the nuclei were stained with DAPI (blue). **C** the expression of meiosis-related genes detected by RT-qPCR at 12 days of differentiation, n = 3, *p < 0.05 when compared with VPA and # *p* < 0.05 when compared with VPA + VC

In the previous studies, very few cells went through meiosis to generate haploid cells during SSCLC induction, and these haploid cells were suggested to be putative spermatids as they expressed spermatids-specific markers (Acrosin and PRM1) [11, 16]. We examined whether meiotic or haploid cells existed in differentiated cells when using our developed protocol. The expression of meiotic gene *SYCP3* (restricted to spermatocytes) slightly increased from 12 to 14 days of differentiation, but another gene *STRA8* (restricted to differentiated spermatogonium) hardly expressed (Fig. 2C). At 12 days of differentiation, the expression of *STRA8* and *SYCP3* was higher in VPA and VPA + VC groups than that in VC and 0.5 (VPA + VC) groups (Fig. 3C). We used PI to stain cell DNA and detected the percentage of haploid cells by flow cytometry. Few haploid cells (< 2%) were generated at 12 days of differentiation in VC and 0.5 (VPA + VC) groups (Fig. 3A). Interestingly, we observed over 5% haploid cells in VPA and VPA + VC groups (Fig. 3A). Immunofluorescence detected SYCP3^+^ cells representing meiotic cells, and Acrosin^+^ or TNP1^+^ (a spermatids-specific marker) cells representing haploid cells in 0.5 (VPA + VC) group at 12 days of differentiation, but these cells accounted for a very small fraction of differentiated cells (Fig. 3B). These results indicated that cells might go through meiosis in our developed SSCLC induction system and a relatively high concentration of VPA induced further differentiation.

### Transcriptome analysis of hiPSCs and cells differentiated from hiPSCs

**Fig. 4:**
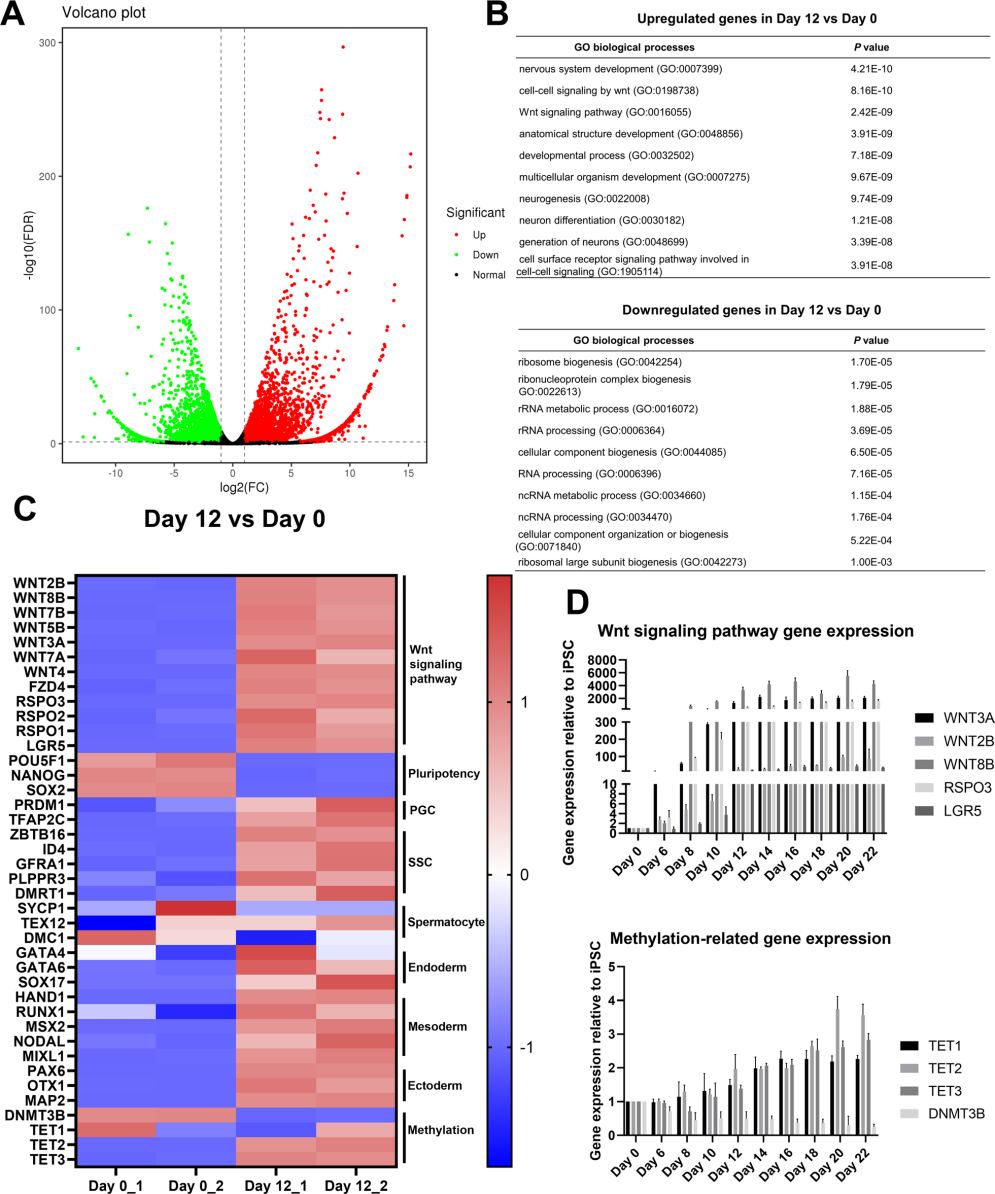
Transcriptome analysis of hiPSCs and cells at 12 days of differentiation. **A** The volcano plot of DEGs between hiPSCs (Day 0) and cells at 12 days of differentiation (Day 12), red dots represented significantly upregulated genes, green dots represented significantly downregulated genes and black dots represented not significantly changed genes. **B** GO terms (biological process) of upregulated and downregulated genes, respectively. **C** Heatmap of significantly changed genes between Day 0 and Day 12 cells. **D** The expression of Wnt signaling pathway genes and methylation-related genes at different days of differentiation detected by RT-qPCR, n = 3

We compared the transcriptome of hiPSCs (Day 0) and cells at 12 days of differentiation (Day 12) to investigate changes in gene expression during SSCLC induction. There were 7702 DEGs in Day 12 cells compared with Day 0 cells, 4489 genes and 3212 genes of which were upregulated and downregulated, respectively (Fig. 4A). GO analysis had significantly enriched upregulated genes into development (e.g. nervous system development, anatomical structure development and multicellular organism development) and Wnt signaling pathway, and enriched downregulated genes into ribosome biogenesis and RNA processing (Fig. 4B). Several GO terms were related to nervous system and neurogenesis might because that GDNF has important roles (maintaining several neuronal populations) in the central nerves system and VPA promotes neural differentiation [34]. SSCLC induction process generated a mix of different types of cells. We found that at 12 days of differentiation, Wnt signaling pathway genes, primordial germ cell (PGC)-related genes, SSC-related genes, endodermal genes, mesodermal genes and ectodermal genes were upregulated, but pluripotent genes were downregulated (Fig. 4C). Spermatocyte-related genes were hardly detected (Fig. 4C). Moreover, *DNMT3B* expression was reduced and *TETs* were upregulated, and these genes have roles in DNA methylation and demethylation (Fig. 4C). RT-qPCR had confirmed increased expression of genes in Wnt signaling pathway and *TETs* and decreased expression of *DNMT3B* at 12 days of differentiation (Fig. 4D), indicating that SSCLC induction might involve Wnt signaling pathway activation and the change of genome methylation.

### VPA affected histone modification during differentiation

A previous study reported genome-wide increase in H3K9ac in mouse ESCs after treated with 0.5 mM VPA and H3K9ac was correlated with gene expression [35]. In early human germ cell development, increased H3K9ac is accompanied with decreased H3K27me3 [36]. We explored whether these two histone marks were changed during SSCLC induction. Western blot results showed increased H3K9ac and decreased H3K27me3 in cells at 12 days of differentiation using our developed protocol (Additional file 6: Fig. S5 A). The same results were observed in VPA group, suggesting that VPA could affect histone modifications (Additional file 6: Fig. S5 A). We also determined the gene expression of class I HDACs (*HDAC1*, *HDAC2* and *HDAC3*) and *KDM6B* (H3K27me3 demethylase) in cells at different days of differentiation (Additional file 6: Fig. S5 B). The expression of *HDAC2* was decreased while the expression of *KDM6B* was increased along with differentiation, which might lead to altered histone modifications.

### Differentiation of hiPSCs from NOA patients into SSCLCs

**Fig. 5:**
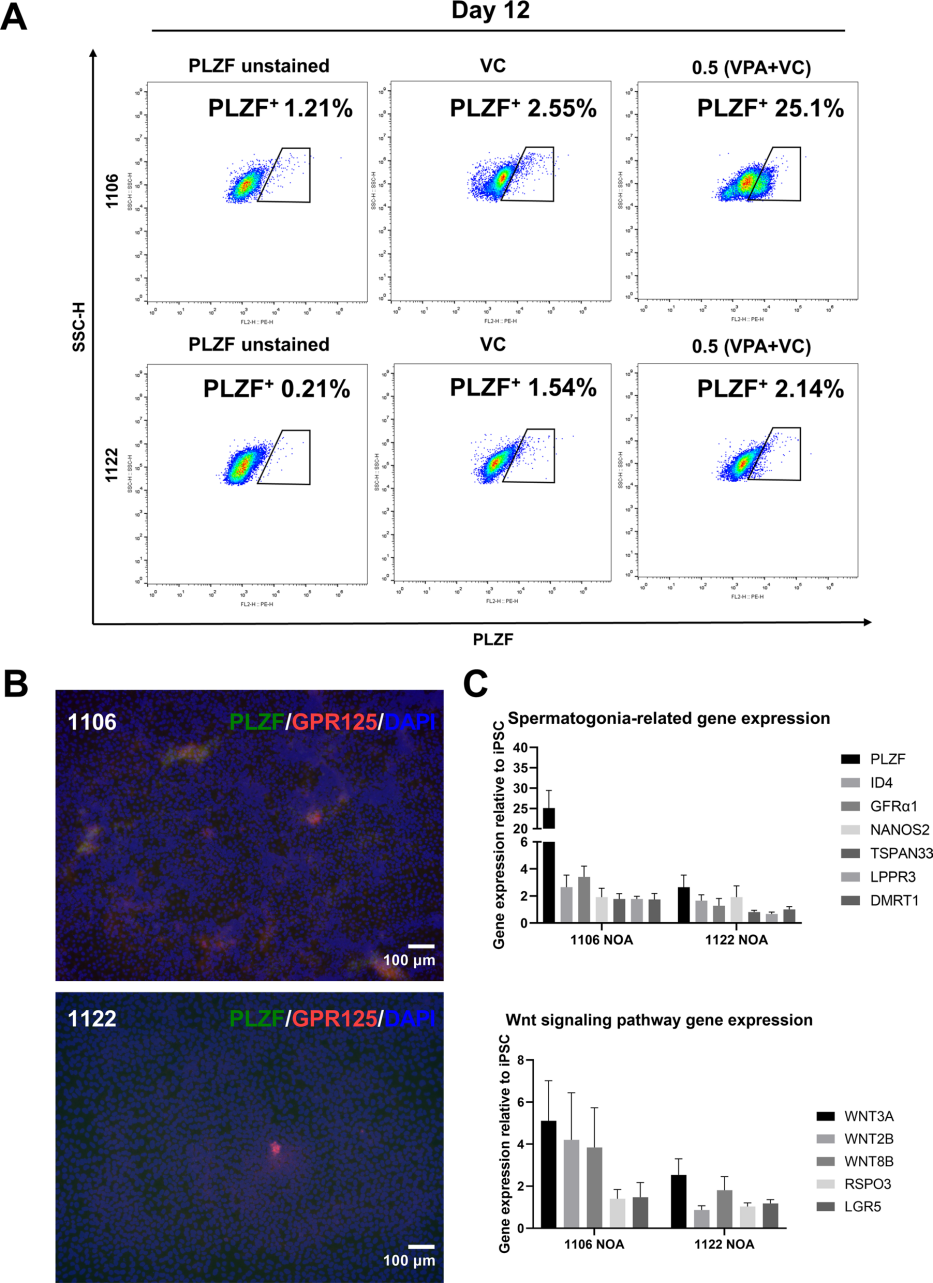
Differentiation of hiPSCs from NOA patients into SSCLCs. **A** The percentage of PLZF^+^ cells of 1106 and 1122 NOA patients at 12 days of differentiation detected by flow cytometry. **B** Immunostaining of PLZF (green) and GPR125 (red) at 12 days of differentiation, and the nuclei were stained with DAPI (blue). **C** The expression of genes related to SSC and Wnt signaling pathway at 12 days of differentiation detected by RT-qPCR, n = 3

We next differentiated two hiPSC lines (1106 and 1122 hiPSC lines), which were from unrelated NOA patients with unknown cause and testicular histology, into SSCLCs using different protocols. These two cell lines showed different responses to different protocols. At 12 days of differentiation, 1106 hiPSCs could be differentiated into a higher percentage of SSCLCs using our developed protocol (Fig. 5A). 1122 hiPSCs were hardly to be differentiated into SSCLCs using different protocols (Fig. 5A), indicating that this patient might have serious abnormalities of SSCs and the disease cause was associated with SSCs. Immunofluorescence detected few PLZF^+^/GPR125^+^ cells in 1106 group at 12 days of differentiation and only few GPR125^+^ cells were observed in 1122 group (Fig. 5B). RT-qPCR results also showed low expression of SSC-related genes and Wnt signaling pathway genes in 1106 group (Fig. 5C). Compared with the normal hiPSC line differentiated at 12 days (Figs. 2A, C, 4D), the SSCLC induction efficiency and the expression of genes related to SSC and Wnt signaling pathway were reduced in NOA groups (Fig. 5A, C), implying low SSCLC induction efficiency might be associated with the defects of Wnt signaling pathway activation.

## Discussion

In this study, we showed that VPA robustly promoted the differentiation of human pluripotent stem cells into SSCLCs in the presence of human GDNF and b-FGF. The combination of low concentrations of VPA and VC in the induction medium achieved the highest SSCLC induction efficiency at 12 days of differentiation. A high concentration of VPA also elevated the expression of meiosis-related genes and generated more haploid cells. RNA-seq analysis revealed that the SSCLC induction involved the upregulated genes in Wnt signaling pathway, and low SSCLC induction efficiency of NOA hiPSCs might be associated with inactivated Wnt signaling pathway.

SSCs have ability to self-renew and give rise to meiotic spermatocytes that finally generate spermatozoa [37]. The abnormalities of SSCs can lead to the failure of spermatogenesis and result in azoospermia. In humans, genes associated with azoospermia have been largely identified in recent years [38, 39]. The association between genetic cause and azoospermia is generally validated by mouse models because human germ cells are difficult to derive and culture in vitro and used as cell models. In vitro modeling system is a more widely used and effective way in screening and validating genes associated with diseases, but the in vitro systems were not well established for the study of male infertility [39]. Our developed SSCLC induction protocol successfully and efficiently differentiated hiPSC lines and H1 ESCs into SSCLCs. Moreover, compared with the normal hiPSC line, the NOA hiPSC lines were differentiated into less SSCLCs. The SSCLC induction system could be used as an in vitro model to reflect possible abnormalities in SSCs of NOA patients. hiPSC lines established from individuals carry the specific genetic variants [39], combined with the developed SSCLC induction protocol, we can derive direct evidence to assess the effects of genetic variants identified in NOA patients on SSCs. We also observed that hiPSC lines of NOA patients showed different responses to different SSCLC induction protocols. Although our developed SSCLC induction protocol can be used as a cell model to facilitate the study of male infertility, using different protocols to test SSCLC induction efficiency of a NOA hiPSC line can provide more reliable evidence to understand the condition of SSCs in NOA testes.

The role of VPA in promoting differentiation has been linked to its effect on epigenetic modification [26, 28, 33]. As a class I HDAC inhibitor, VPA could affect epigenetic marks and chromatin structure to alter gene expression [19]. VPA affects methylation of DNA and histones and demethylation of histones in several cell types [19]. VC also has effect on reprogramming of gene expression through demethylation of 5-mC in DNA and lysine demethylation of histones [40]. We noticed that cells differentiated with VPA exhibited increased H3K9ac and decreased H3K27me3, and the expression of *HDAC2* and *KDM6B* was downregulated and upregulated, respectively. These results indicated that altered epigenetic modifications were involved in SSCLC induction. The highest SSCLC induction efficiency in the presence of low concentrations of VPA and VC might be associated with their effects on altering epigenetic modifications.

The increased SSCLC induction efficiency was not only reflected by the higher percentage of PLZF^+^ cells, but also the increased expression of various SSC-related genes. However, whether the increased SSCLC induction efficiency was directly related to altered epigenetic modifications still needs further studies to confirm, because VPA could stimulate signaling pathways or transcript factors to enhance target gene expression rather than directly modulating gene expression through epigenetic regulation [41–44]. Studies have reported that VPA upregulated Wnt signaling pathway genes during neural differentiation and neurogenesis [41, 44], and this effect might be the result of VPA induced beta-catenin and phospho-GSK3 or altered demethylation of Wnt-activators [41, 44, 45]. In this study, RNA-seq and RT-qPCR had confirmed the upregulated Wnt signaling pathway genes along with SSCLC induction. The expression of Wnt signaling pathway genes was decreased in cells of NOA groups with low SSCLC induction efficiency. The role of Wnt signaling pathway in human and mouse PGC specification and mouse SSC maintenance has been revealed in previous studies [46–51]. Our study proposed the critical role of Wnt signaling pathway in human SSCLC induction. The depletion of SSCs in azoospermia testes might be associated with inactivated Wnt signaling pathway.

We showed that VPA was able to promote the induction of SSCLCs. The effects of VPA on SSCLC induction seemed to be dose-dependent. When used alone, high concentration of VPA was more effective to induce SSCLCs than low concentration of VPA. The addition of low concentration of VC to the medium containing low concentration of VPA largely boosted the SSCLC induction efficiency. Moreover, compared with the combination of low concentrations of VPA and VC, high concentration of VPA and the combination of high concentrations of VPA and VC led to less SSCLCs but more haploid cells, indicating that high concentration of VPA induced further differentiation. These results suggested that the concentrations of VPA and VC could be adjusted with the purpose of experiment. The combination of low concentrations of VPA and VC was an ideal protocol to obtain a high percentage of SSCLCs.

The current study was an improvement of the existing SSCLC induction protocols, which aimed to promote the SSCLC induction efficiency. Based on existing studies, the global transcript dynamics of SSCLCs were different from in vivo isolated human SSCs [16], and SSCLCs were not mature and difficult to complete further differentiation in vivo [52]. Our study had showed that a high concentration of VPA could induce further differentiation and generate more haploid cells. VPA alone, or VPA combined with other small molecules (e.g. VC and retinoic acid) might be a new option to stimulate differentiation of SSCLCs and derive haploid cells. For the wider application in used as disease model and developing therapy, there must be further research to compare the similarity between SSCLCs and human SSCs, and to confirm whether SSCLCs can be further differentiated.

## Conclusions

VPA robustly promoted the differentiation of human pluripotent stem cell lines into SSCLCs, and the combination of low concentrations of VPA and VC was most effective to induce SSCLCs. The high concentration of VPA could induce further differentiation and generate more haploid cells during SSCLC induction. Differentiation of hiPSCs into SSCLCs involved the upregulated Wnt signaling pathway genes and epigenetic changes. hiPSCs from NOA patients showed decreased SSCLC induction efficiency and Wnt signaling pathway gene expression, suggesting that SSC depletion in azoospermia testes might be associated with inactivation of Wnt signaling pathway. Our developed SSCLC induction protocol provides a reliable tool and model to study human germ cell development and male infertility.

## Supplementary Information

**Additional file 1: Table S1.** Antibodies used in this study. **Table S2** Primers used in this study.

**Additional file 2: Fig. S1**. Differentiation of H1 ESCs into SSCLCs using different SSCLC induction medium. The percentage of PLZF^+^ cells was detected by flow cytometry at 12 days of differentiation.

**Additional file 3: Fig. S2**. Differentiation of hiPSCs into SSCLCs using SSCLC induction medium containing different concentrations of GDNF and b-FGF. A, immunostaining of PLZF (green) of differentiated cells using SSCLC induction medium containing different concentrations of GDNF and b-FGF at 12 days of differentiation, and the nuclei were stained with DAPI (blue). The GF group was equivalent to the VC group. B, the percentage of PLZF^+^ cells was detected by flow cytometry at 12 days of differentiation.

**Additional file 4: Fig. S3**. Percentage of apoptosis cells at 5 days of differentiation using SSCLC induction medium containing different concentrations of VPA and/or VC. Apoptosis cells were stained with PI and Annexin V and were detected by flow cytometry, n=3, * p<0.05 when compared with VC.

**Additional file 5: Fig. S4**. Percentage of SSCLCs at 4 and 8 days of differentiation using different SSCLC induction medium.

**Additional file 6: Fig. S5**. Altered histone modifications during SSCLC induction. A, the histone marks H3K9ac and H3K27me3 of hiPSCs and cells at 12 days of differentiation determined by western blot. B, the expression of HDACs and KDM6B at different days of differentiation detected by RT-qPCR, n=3.

## Data Availability

The datasets supporting the conclusions of this article are available in the SRA database, with unique accession code PRJNA756148. All other relevant data are included in this article.

## Abbreviations

SSC: Spermatogonial stem cell
NOA: Non-obstructive azoospermia
SCOS: Sertoli cell-only syndrome
WES: Whole exome sequencing
hiPSC: Human induced pluripotent stem cell
hESC: Human embryonic stem cell
SSCLC: Spermatogonial stem cell-like cell
VC: Vitamin C
VPA: Valproic acid
FDR: False discovery rate
DEG: Differentially expressed gene
GO: Gene ontology
PGC: Primordial germ cell
