# Supplementary material for: Valproic acid promotes the in vitro differentiation of human pluripotent stem cells into spermatogonial stem cell-like cells
Source: Stem Cell Res Ther. 2021 Oct 29;12:553. doi: 10.1186/s13287-021-02621-1 (PMC8555208; doi:10.1186/s13287-021-02621-1)
Supplement: Supplementary file 1 — Additional file 1: Table S1. Antibodies used in this study. Table S2 Primers used in this study. [file 13287_2021_2621_MOESM1_ESM.docx]

**Additional File Table 1 Antibodies used in this study**

| **Antibodies** | **Vendor** | **Dilution rate** |
| --- | --- | --- |
| **Primary antibodies** |  |  |
| PLZF mouse monoclonal antibody | Santa Cruz | 1:150 |
| GPR125 rabbit polyclonal antibody | GeneTex | 1:150 |
| GFRα1 rabbit polyclonal antibody | Boster | 1:150 |
| Acrosin rabbit polyclonal antibody | Absin | 1:150 |
| TNP1 rabbit polyclonal antibody | Proteintech | 1:150 |
| SYCP3 mouse monoclonal antibody | Santa Cruz | 1:100 |
| PIWIL2 rabbit polyclonal antibody | Abcam | 1:150 |
| VASA mouse monoclonal antibody | Santa Cruz | 1:150 |
| H3K9ac rabbit polyclonal antibody | Cell Signaling Technology | 1:800 |
| H3K27me3 rabbit polyclonal antibody | Cell Signaling Technology | 1:800 |
| Histone H3 rabbit polyclonal antibody | Abclonal | 1:1000 |
| PLZF mouse monoclonal antibody-PE | Invitrogen | 1:200 |
| **Secondary antibodies** |  |  |
| CoraLite594 conjugate Goat anti-Mouse IgG | Proteintech | 1:400 |
| CoraLite594 conjugate Goat anti-Rabbit IgG | Proteintech | 1:400 |
| CoraLite488 conjugate Goat anti-Mouse IgG | Proteintech | 1:400 |
| Peroxidase-conjugated Affinipure Goat Anti-Rabbit IgG | Proteintech | 1:5000 |

**Additional File Table 2 Primers used in this study**

| **Primers** | **Sequences** |
| --- | --- |
| PLZF | GAGATCCTCTTCCACCGCAAT/ CCGCATACAGCAGGTCATC |
| ID4 | TCCCGCCCAACAAGAAAGTC/ CTGCAGGTCCAGGATGTAGTC |
| GFRα1 | TGGAGCACATTCCCAAAGGG/ AGCATTCCGTAGCTGTGCTT |
| NANOS2 | TCGCAAGTCGGGGTCAAAG/ CTGGTGTGAGGAGTAGACGTG |
| TSPAN33 | AGCCCGCTGGTGAAATACCT/ TAGGGCTGCTTCTGCATGCTT |
| LPPR3 | GCTATGACCGCACTCTCTCC/ ACAGTACAACATGCCCTCGG |
| DMRT1 | CCAGCCGTCTCTGTTTCCTT/ CCCCAGAAGCAGAATCAGCA |
| STRA8 | ACTCTCAGTCTGATCTCATAGCC/ TACCAAGGGGAGGAACCATTC |
| SYCP3 | TTTGTTTCAGCAGTGGGATT/ TTCCGAACACTTGCTATCTC |
| OCT4 | GACAGGGGGAGGGGAGGAGCTAGG/ CTTCCCTCCAACCAGTTGCCCCAAAC |
| SOX2 | GGGAAATGGGAGGGGTGCAAAAGAGG/ TTGCGTGAGTGTGGATGGGATTGGTG |
| NANOG | ACCAGTCCCAAAGGCAAACA/ TCTGCTGGAGGCTGAGGTAT |
| WNT3A | TGCTGGACAAAGCTACCAGG/ CGAGACACCATCCCACCAAA |
| WNT2B | CCGAGAGTGTCAGCACCAAT/ CGCGAGTAATAGCGTGGACT |
| WNT8B | ACAGCTGGTCGGTGAACAAT/ CTGCCACACTGCTGGAGTAA |
| RSPO3 | ATACATCGGCAGCCAAAACG/ CTTCCAACCCTTCTGGGCAA |
| LGR5 | CATCAGCTATGTGCCCCCAA/ TGTGGAGCCCATCAAAGCAT |
| TET1 | ACAACCAAGGGAGCCAACAA/ ATTTGGCTACGACCAGTGGG |
| TET2 | CCTTCTCTCTCTGGGCTCCT/ AGCTCTGGATTTTCAGGCCC |
| TET3 | TTATGACTTCCCTCAGCGCC/ ACCACACCGTTTCCGTTTCT |
| DNMT3B | GGAGAAAGCTAGGGTGCGAG/ AATTTCCTACTGCCTGCACGA |
| HDAC1 | TGCTAAAGTATCACCAGAGGGT/ TGGCCTCATAGGACTCGTCA |
| HDAC2 | GCCACTGCCGAAGAAATGAC/ TCCAGCCCAATTAACAGCCA |
| HDAC3 | TGCTGAACCATGCACCTAGT / CCCTTTTAAACCTCCCCAGC |
| KDM6B | ACATGGCAGTAGTTCTGGGC/ GCCTCCTCACTATCGTGCTC |
