# Supplementary figures and images for: Valproic acid promotes the in vitro differentiation of human pluripotent stem cells into spermatogonial stem cell-like cells
Source: Stem Cell Res Ther. 2021 Oct 29;12:553. doi: 10.1186/s13287-021-02621-1 (PMC8555208; doi:10.1186/s13287-021-02621-1)

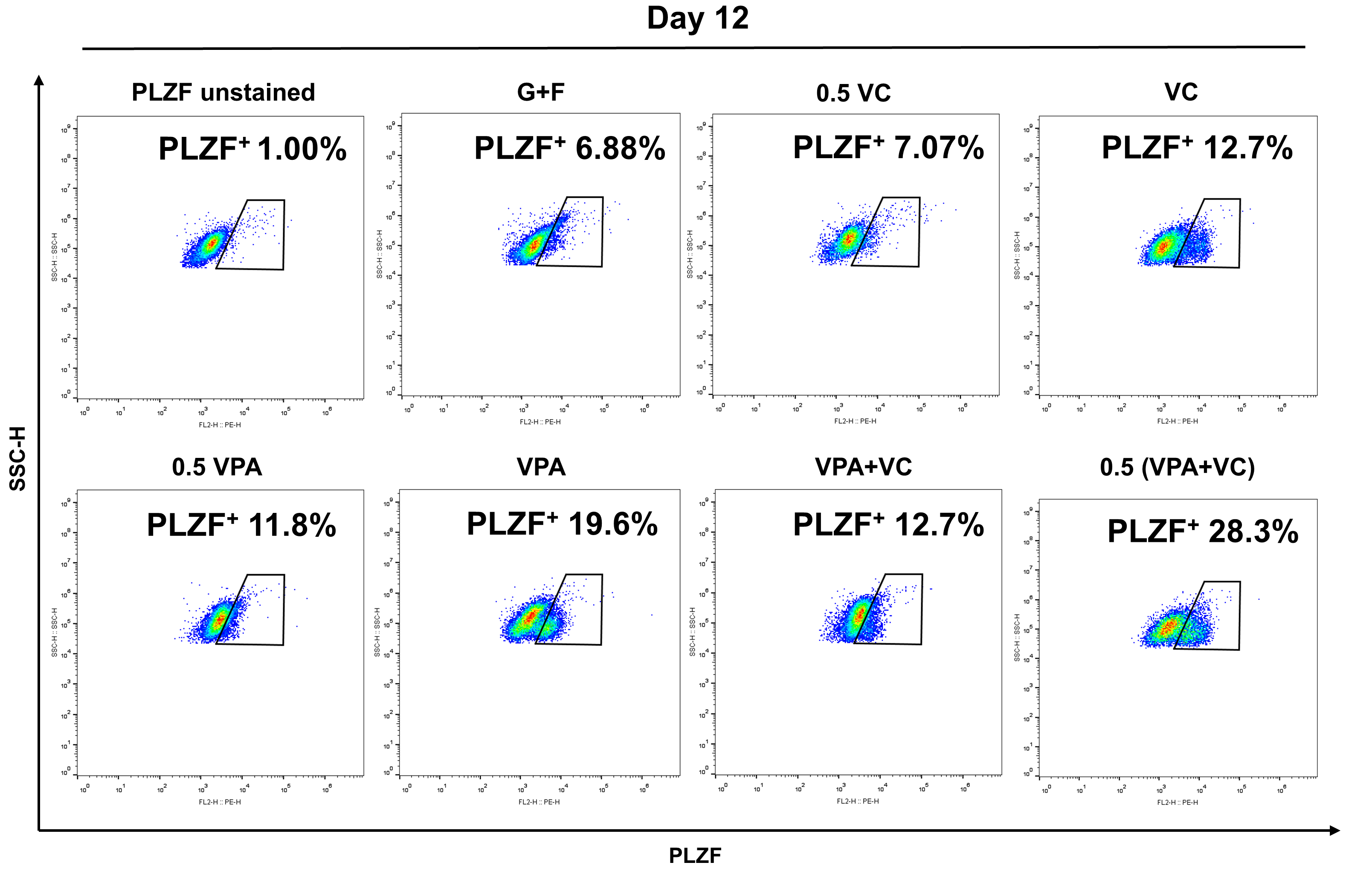

Supplement: Supplementary file 2 — Additional file 2: Fig. S1. Differentiation of H1 ESCs into SSCLCs using different SSCLC induction medium. The percentage of PLZF+ cells was detected by flow cytometry at 12 days of differentiation. [file 13287_2021_2621_MOESM2_ESM.tif]

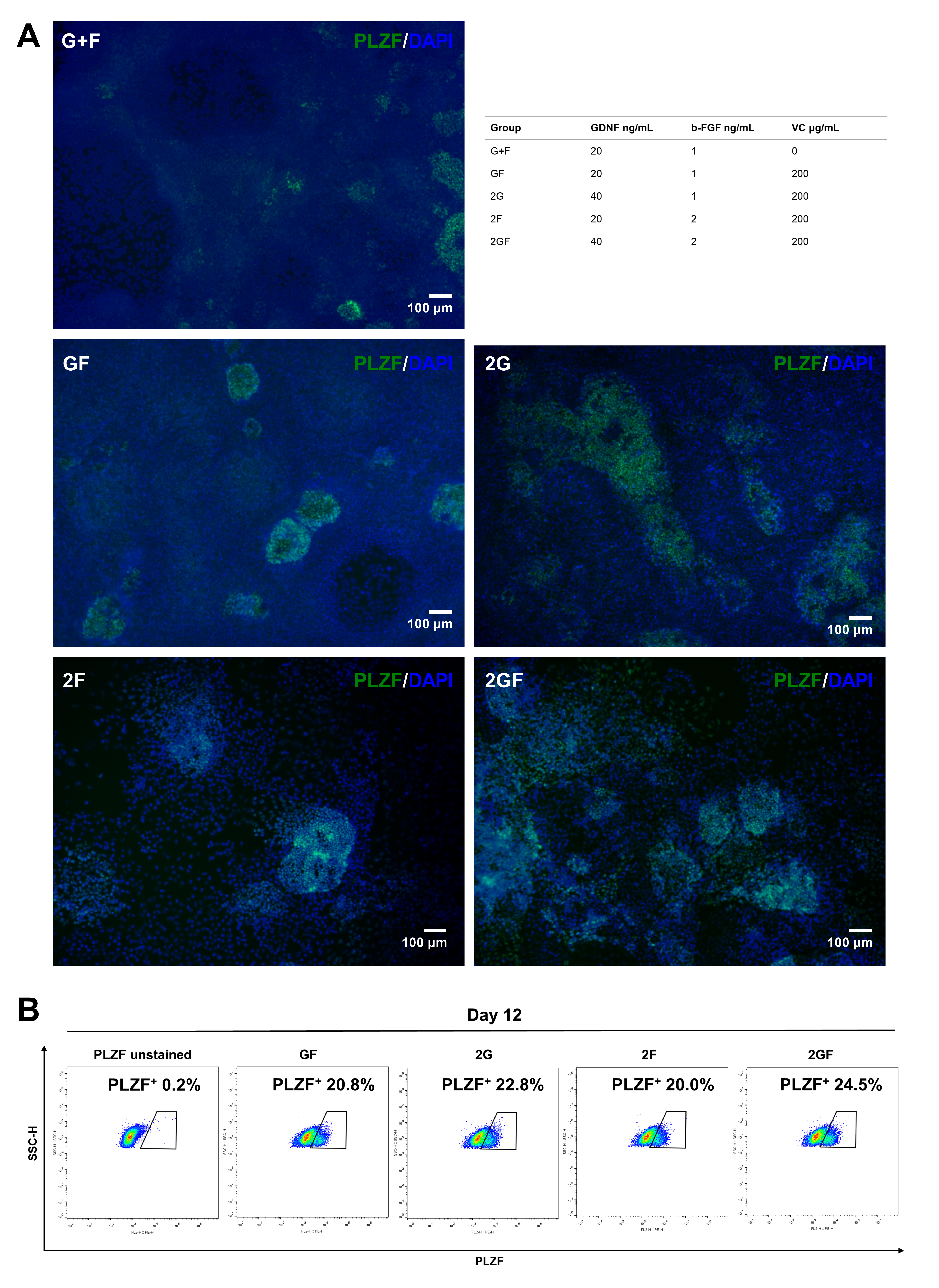

Supplement: Supplementary file 3 — Additional file 3: Fig. S2. Differentiation of hiPSCs into SSCLCs using SSCLC induction medium containing different concentrations of GDNF and b-FGF. A, immunostaining of PLZF (green) of differentiated cells using SSCLC induction medium containing different concentrations of GDNF and b-FGF at 12 days of differentiation, and the nuclei were stained with DAPI (blue). The GF group was equivalent to the VC group. B, the percentage of PLZF+ cells was detected by flow cytometry at 12 days of differentiation. [file 13287_2021_2621_MOESM3_ESM.tif]

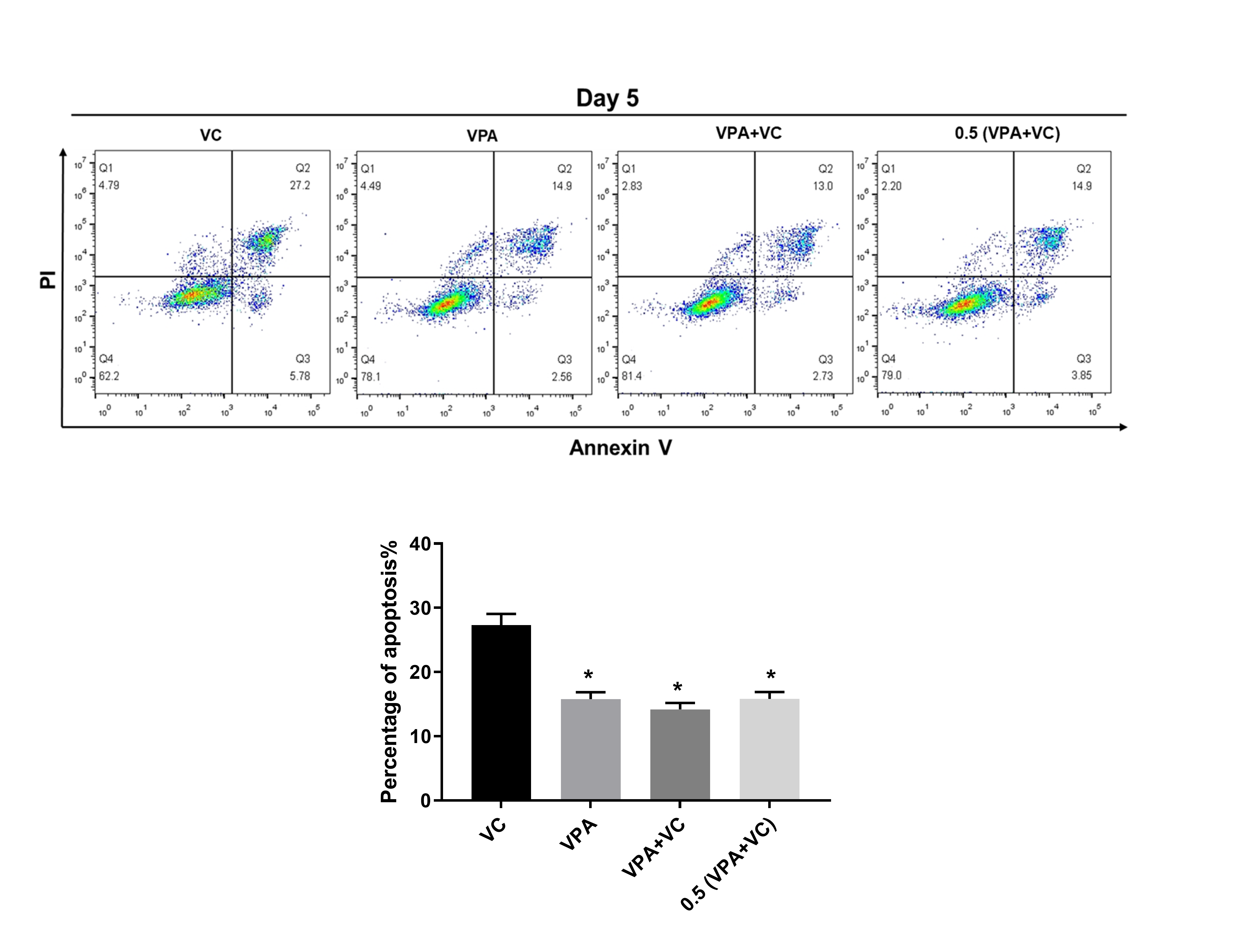

Supplement: Supplementary file 4 — Additional file 4: Fig. S3. Percentage of apoptosis cells at 5 days of differentiation using SSCLC induction medium containing different concentrations of VPA and/or VC. Apoptosis cells were stained with PI and Annexin V and were detected by flow cytometry, n=3, * p<0.05 when compared with VC. [file 13287_2021_2621_MOESM4_ESM.tif]

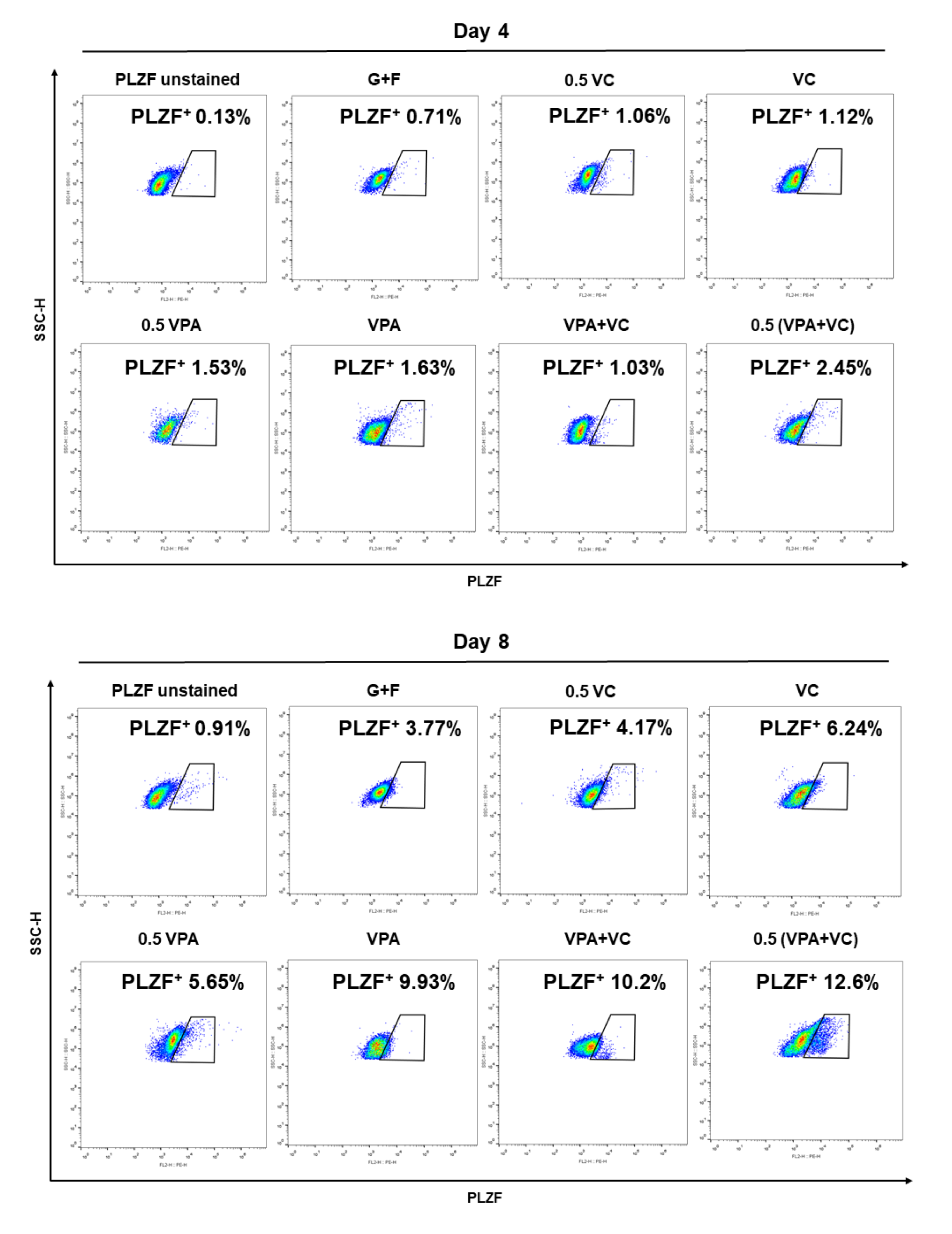

Supplement: Supplementary file 5 — Additional file 5: Fig. S4. Percentage of SSCLCs at 4 and 8 days of differentiation using different SSCLC induction medium. [file 13287_2021_2621_MOESM5_ESM.tif]

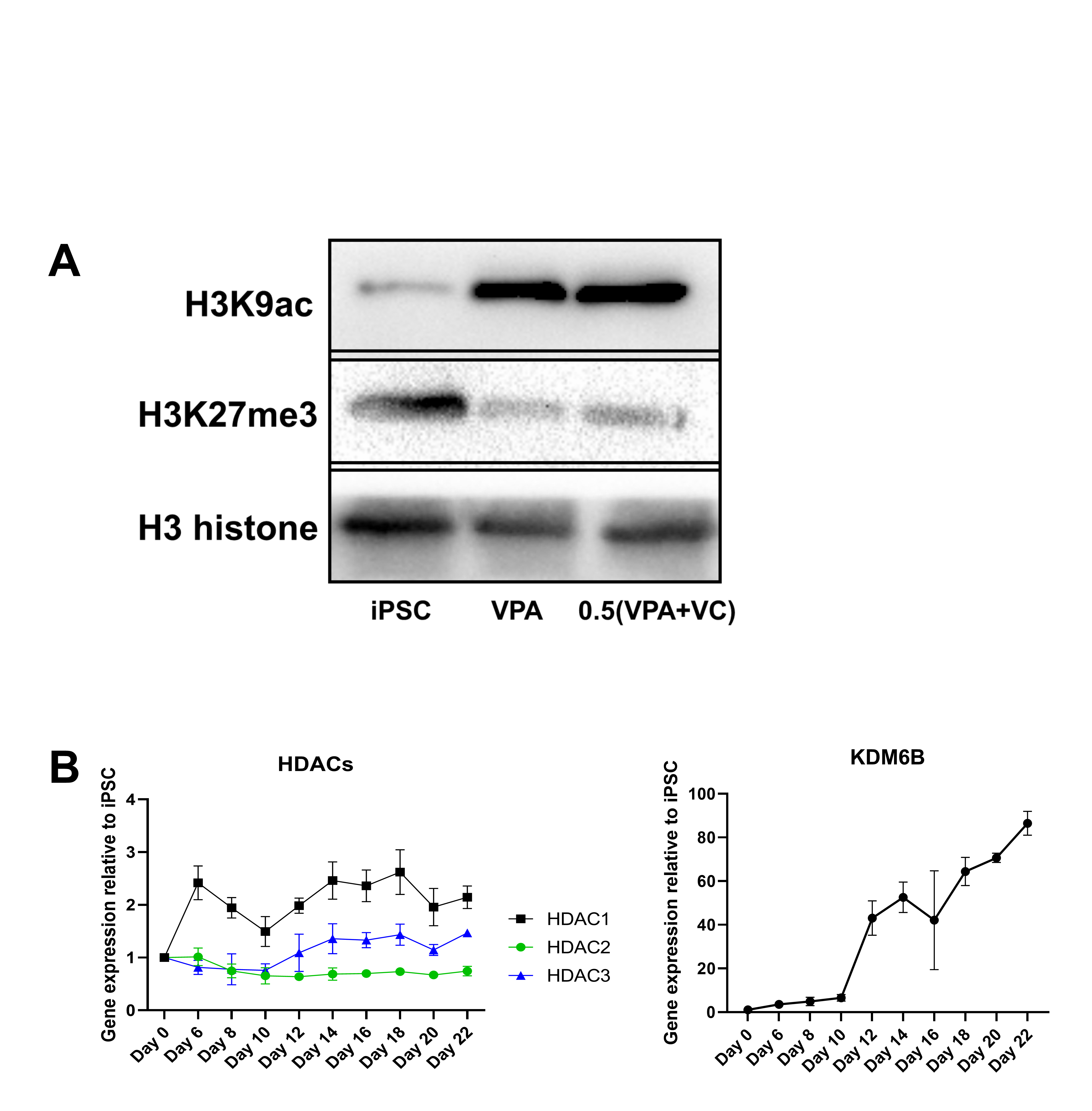

Supplement: Supplementary file 6 — Additional file 6: Fig. S5. Altered histone modifications during SSCLC induction. A, the histone marks H3K9ac and H3K27me3 of hiPSCs and cells at 12 days of differentiation determined by western blot. B, the expression of HDACs and KDM6B at different days of differentiation detected by RT-qPCR, n=3. [file 13287_2021_2621_MOESM6_ESM.tif]
